# Development and Validation of a Nomogram for the Prediction of Inguinal Lymph Node Metastasis Extranodal Extension in Penile Cancer

**DOI:** 10.3389/fonc.2021.675565

**Published:** 2021-06-17

**Authors:** Chong Wu, Zaishang Li, Shengjie Guo, Fangjian Zhou, Hui Han

**Affiliations:** ^1^ Department of Urology, Sun Yat-sen University Cancer Center, Guangzhou, China; ^2^ State Key Laboratory of Oncology in South China, Guangzhou, China; ^3^ Collaborative Innovation Center of Cancer Medicine, Guangzhou, China; ^4^ Department of Urology, Shenzhen People’s Hospital, The Second Clinic Medical College of Jinan University, Shenzhen, China; ^5^ Department of Urology, First Affiliated Hospital of Southern University of Science and Technology, Shenzhen, China; ^6^ Minimally Invasive Urology of Shenzhen Research and Development Center of Medical Engineering and Technology, Shenzhen, China

**Keywords:** risk assessment, risk model, nomogram, extranodal extension, penile cancer

## Abstract

**Purpose:**

To determine whether a clinicopathologic and laboratory-based nomogram is capable of predicting the risk of lymph node extranodal extension (ENE) in patients with penile cancer.

**Materials and Methods:**

From June 2006 to January 2021, 234 patients who underwent bilateral inguinal lymph node dissection (ILND) surgery were included in the analysis. A Lasso regression model was utilized to select the most useful predictive features from among 46 laboratory variables. Then, a logistic regression analysis was used to develop the prediction model. Calibration curves, concordance index (C-index) and Areas under the receiver-operating characteristic curves (AUCs) were performed to evaluate the performance of the nomogram. We also investigated model fit using changes in Akaike Information Criteria (AICs). Decision curve analyses (DCAs) were applied to assess the clinical usefulness of this nomograms. Its internal validation was confirmed.

**Results:**

Among the 234 patients, 53 were confirmed to have ENE. The platelet-lymphocyte ratio (PLR) and Squamous cell carcinoma antigen (SCC-Ag) were significantly associated with ENE (P<0.05). The individualized prediction nomogram, including the PLR, SCC-Ag, lymphovascular invasion (LVI), and pathologic tumor stage(pT-stage), showed good discrimination, with a C-index of 0.817 (95% CI, 0.745 to 0.890) and good calibration. Clinical-laboratory nomogram (AIC, 180.034) become the best-fitting model. DCA findings revealed that the clinical-laboratory nomogram was more clinically useful than the pT-stage or tumor grade.

**Conclusions:**

This study presents a clinicopathologic and laboratory-based nomogram that incorporates PLR, SCC-Ag, lymphovascular invasion (LVI), and pT-stage, which can be conveniently utilized to facilitate the individualized prediction of lymph node metastasis ENE in patients with penile cancer.

## Introduction

Penile squamous cell carcinoma (PSCC) is an uncommon malignancy, describing 0.4% to 0.6% of all malignant disease among men in the United States and Europe (1). Its incidence is higher among men in developing regions (2).

One of the most unfavorable prognostic factors in penile cancer for poor prognosis is lymph node metastasis (LNMs). Extranodal extension (ENE) is defined as extension of the tumor through the lymph node capsule into the perinodal fibrous-adipose tissue and is an independent adverse prognostic factor in PSCC (3, 4).

In the 8th American Joint Committee on Cancer Tumor Node Metastasis (AJCC TNM) staging1, both ENE and pelvic lymph node metastasis (PLNM) are staged as pN3, suggesting that ENE is an adverse pathological characteristic. Johnson and colleagues (5) reported that 5-year survival is reduced by approximately half with lymph node involvement(LNI). Extranodal extension (ENE) of LNMs portends an even worse prognosis. ENE has similarly been implicated in worse outcomes in carcinoma of the bladder, breast, pancreas, stomach, and cervix (6–8).

According to the National Comprehensive Cancer Network (NCCN) guidelines, patients who are pathologically diagnosed with ENE should receive neoadjuvant therapies, and it is reasonable to give 4 courses of (Paclitaxel + Ifosfamide + Cisplatin) TIP in the adjuvant setting if it was not given preoperatively. Nevertheless, the prognosis of the disease remains poor due to a high rate of recurrence. Pelvic lymph node dissection (PLND) should be considered at the time or following inguinal lymph node dissection (ILND) in patients with ≥2 positive inguinal nodes on the ipsilateral ILND site or in the presence of ENE on final pathologic review.

From the above, ENE is an extremely important feature, especially for predicting the outcomes of treatment. In this study, patients who underwent ILND were included, and an individualized prediction model was established and validated.

## Materials and Methods

### Patient Population

The ethics committee of Sun Yat-Sen University Cancer Hospital approved the retrospective data analysis. We retrospectively reviewed 234 patients who underwent bilateral ILND for curative purposes from June 2006 to January 2021. To be eligible for analysis, patients must have met the following criteria: (i) PSCC was their primary disease, (ii) Immediate or delayed ILND, (iii) detailed clinical and pathologic data available, and (iv) no known distant metastasis.

In all patients, variables extracted from their medical records included age, chronic disease (hypertension, heart disease and diabetes), surgical management of the primary tumor (including partial penectomy, lesionectomy and phallectomy), immediate or delayed ILND, perineural invasion (PNI), lymphovascular invasion (LVI), pT-stage, TNM stage, tumor grade, unilateral or bilateral inguinal LNM, number of LNMs and ENE status. Patients were divided into no LNM group, 1 LNM group, 2 LNMs group, 3 LNMs group, and ≥4 LNMs group depend on LNMs status. Laboratory tests were performed within 1 week before the surgery.

### Laboratory Tests

Serum specimens were collected before the bilateral inguinal lymph node dissections. Various routine blood indexes, routine biochemical tests (33 source indicators) and coagulation tests of hemostasis were tested by LABOSPECT 008 AS and Sysmex CS5100, respectively. The SCC-Ag was detected by immunodetection (Cobas e801).

### Construction and Validation of the Nomogram

We incorporated clinicopathological and laboratory indicators as predicted factors into the design of the nomogram. We used the least absolute shrinkage and selection operator (LASSO) regression with 10-fold cross-validation to select variables that were predictive of ENE. Then, a logistic regression model was adapted to screen out the significant (P<0.05) predictors of ENE from the clinicopathological features and the candidate laboratory indicators. Then, we developed a nomogram to predict the probability of ENE.

Areas under the receiver-operating characteristic curves (AUCs) and Harrell’s C-index was used to describe performance and accuracy of nomogram. Model fitting is conducted using AIC and calibration curve, accompanied by the Hosmer-Lemeshow test. Higher AUCs indicated better discrimination and lower AICs indicated superior model-fitting. The calibration curves were assessed by reviewing the predicted versus actual probabilities. Clinical usefulness and net benefit were estimated with decision curve analysis.

### Statistics

Statistical analyses were performed defining a two-sided P<0.05 as significant. Models, statistics, and figures were prepared using SAS 9.4 software version (Cary, NC) and R 3.5.1 (http://www.cran.r-project.org).

## Results

### Patient Characteristics

A total of 234 PSCC patients (median age: 54.7 years; IQR: 46-64) were eligible, including 79 (33.9%)≤T1, 49 (21.0%) in T2, 99 (42.5%) in T3, and 6 (2.6%) in T4 tumor stage. The clinicopathologic characteristics and treatment option of patients with penile cancer are shown in **Table 1**. Inguinal lymph metastasis occurred in 103 (44.0%) patients, and 53 (22.6%) patients were confirmed to have ENE.

**Table 1:** Clinical characteristics of 234 patients with penile cancer.

Characteristic	No. of patients (%) (n = 234)
Age, yr, median (IQR)	55.0 (45.8-64.0)
pT-stage	
≤pT1	79 (33.9)
pT2	49 (21.0)
pT3	99 (42.5)
pT4	7 (2.6)
pN-stage	
pN0	131 (56.0)
pN1	14 (6.0)
pN2	24 (10.3)
pN3	65 (27.8)
M stage	
M0	234 (100.0)
M1	0 (0.0)
Grade	
G1	123 (52.6)
G2	93 (39.7)
G3	18 (7.7)
No. of positive inguinal lymph nodes	
No positive	131 (56.0)
1 Positive	18 (7.7)
2 Positive	38 (16.2)
3 Positive	13 (5.6)
≥4 Positive	34 (14.5)
Inguinal LNM	
Absent	131 (56.0)
Present	103 (44.0)
Unilateral inguinal LNM	61 (26.1)
Bilateral inguinal LNM	42 (17.9)
Primary tumor surgery and ILND	
Simultaneous	182 (77.8)
Nonsimultaneous	52 (22.2)
Primary tumor surgery	
PPA	180 (76.9)
TPA	41 (17.5)
LC	13 (5.6)
Lymph node ENE^a^	
Positive	53 (22.6)
Negative	181 (77.4)
Adjuvant therapy	
Yes	72 (30.8)
NAC	0 (0)
AC	68 (29.1)
AC + AR	4 (1.7)

RT, radiotherapy; pT-stage, pathology tumor stage; pN-stage, pathology lymph node metastasis stage; IQR, interquartile range; M stage, distant metastasis stage; G, tumor grade; ENE, extranodal extension; AC, adjuvant chemotherapy; AC + AR, adjuvant chemotherapy + radiotherapy; ILND, inguinal lymph node dissection; ILNM, inguinal lymph node metastasis; LC, lesionectomy; LNM, lymph node metastasis; NAC, neoadjuvant chemotherapy; PPA, partial penile amputation; TPA, total penile amputation.

a ENE, Extranodal extension was defined as extension of the tumor through the lymph node capsule into the perinodal fibrous-adipose tissue.

In our study, the T staging and tumor stage of the ENE+ group were significantly higher than those of LNM+ ENE- and patients with no LNM (p<0.0001, <0.001, respectively). In addition, a higher percentage of patients were treated with post-operative adjuvant therapy in the ENE+ group (**Table 2**).

**Table 2:** Patient characteristics and descriptive statistics between different and lymph node status.

Variable	LNM		no LNM n = 131	P
	with ENE^a^n = 53	without ENE^a^n = 50		
Age, yr, median (IQR)	55.0 (46.0-65.0)	58.5 (48.8-70.0)	54.0 (44.0-62.0)	0.0502
pT-stage				p<0.0001
≤pT1	3 (5.7)	12 (24.0)	64 (48.9)	
pT2	14 (26.4)	9 (18.0)	26 (19.8)	
pT3	31 (58.5)	27 (54.0)	41 (31.3)	
pT4	5 (9.4)	2 (4.0)	0 (0.0)	
Grade				p<0.001
G1	16 (30.2)	16 (51.7)	91	
G2	29 (54.7)	29 (39.7)	35	
G3	8 (15.1)	5 (7.7)	5	
Adjuvant therapy				p<0.001
Yes	44 (83.0)	28 (56.0)	0	
NAC	0	0	0	
AC	42 (79.2)	26 (52.0)	0	
AC + AR	2 (3.8)	2 (4.0)	0	

RT, radiotherapy; pT-stage, pathology tumor stage; pN-stage, pathology lymph node metastasis stage; IQR, interquartile range; M stage, distant metastasis stage; G, tumor grade; ENE, extranodal extension.

a ENE, Extranodal extension was defined as extension of the tumor through the lymph node capsule into the perinodal fibrous-adipose tissue.

### Feature Selection

**Figure 1:**
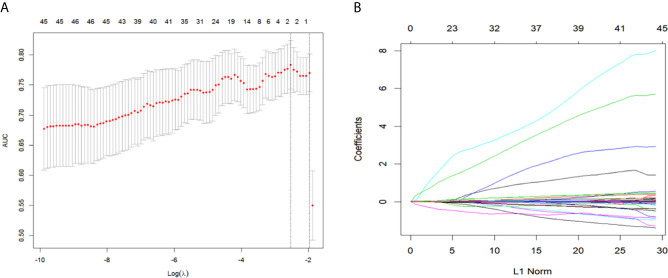
Texture feature selection using the LASSO binary logistic regression model. **(A)** By selecting a 10-fold cross-validation in the LASSO model with minimum standards. The binomial deviance was plotted versus log (λ). Dotted vertical lines were drawn at the optimal λ values based on the minimum criteria and 1 standard error of the minimum standards and the optimal λ was 0.069. **(B)** The LASSO logistic regression algorithm was used to screen out 2 features with non-zero coefficients out of 46 features. LASSO, least absolute shrinkage and selection operator.

**Figure 2:**
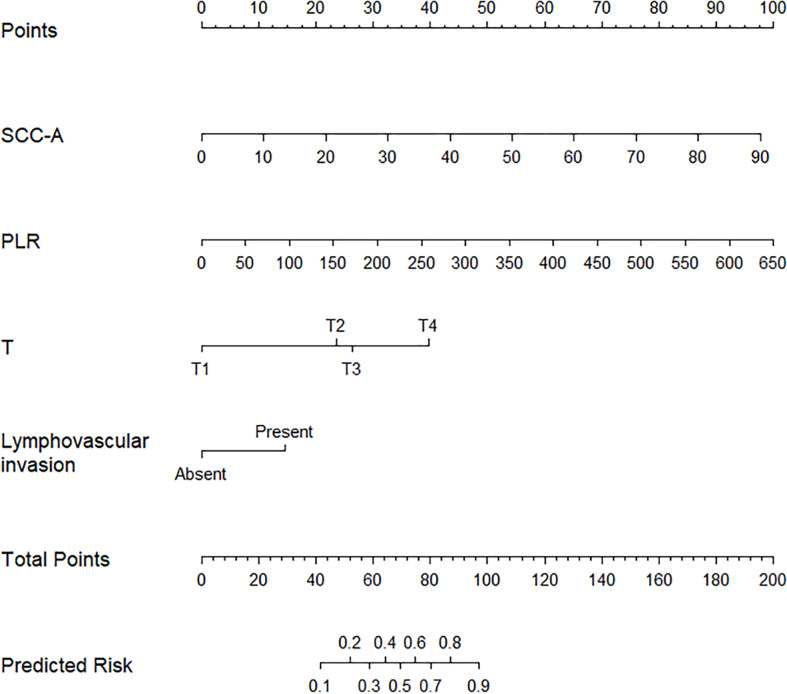
Predicted nomogram for PCCS patients: a line was drawn straight down to predict the risk of ENE. T, Pathology T stage; PLR, platelet-lymphocyte ratio; SCC-Ag, Squamous cell carcinoma antigen.

For the development of the nomogram, we incorporated 46 laboratory tests as predictive features. All of these parameters were reduced to the 2 most useful potential predictors for ENE, with nonzero coefficients in the LASSO regression model (**Figure 1**). As shown in **Figure 2**, the nomogram indicates that platelet-lymphocyte ratio (PLR) has the strongest correlation with ENE and LNM.

### Nomogram Development and Internal Validation

Univariable analysis was performed initially, followed by multivariate analysis (Variables with P < 0.05 on univariate analysis were included in the multivariate model (**Table 3**). Tumor stage, PLR, serum Squamous cell carcinoma antigen (SCC-Ag), tumor grade, PNI and LVI were significant Inguinal lymph node ENE predictors at the initial screening. On multivariate analysis, we found that tumor stage (P=0.006), PLR (P<0.001), SCC-Ag (P=0.001) and LVI (P=0.017) remained independent predictive factors (**Table 3**).

**Table 3:** Univariable and multivariable analyses.

Characteristic	Univariable		Multivariable	
	Odds Ratio (95% CI)	P^a^ value	Odds Ratio (95% CI)	P^a^ value
SCC-Ag	1.123 (1.072-1.177)	<0.001	1.090 (1.035-1.148)	0.001
PLR	1.014 (1.008-1.020)	<0.001	1.012 (1.006-1.019)	<0.001
pT-stage				
≤pT1	reference		reference	
pT2	6.205 (1.909-20.161)	0.002	6.522 (1.716-24.791)	0.006
pT3	9.667 (3.233-28.900)	<0.001	8.077 (2.322-28.103)	0.001
pT4	52.500 (7.680-358.906)	<0.001	23.258 (2.431-222.560)	0.006
Grade				
G1	reference			
G2	3.871 (1.896-7.902)	<0.001		
G3	5.026 (1.682-15.02)	0.004		
PNI	2.424 (1.213-4.845)	0.012		
LVI	5.773 (2.736-12.181)	<0.001	3.205 (1.227-8.371)	0.017

CI, Confidence Interval; OR, odds ratio; PLR, platelet-lymphocyte ratio; SCC-Ag, squamous cell carcinoma antigen; pT-stage, pathology tumor stage; IQR, interquartile range; G, tumor grade; PNI, Perineural invasion; LVI, Lymphovascular invasion.

a P values were calculated using Logistic regression model.

Derived from the four independent predictive factors, a model that incorporated the above predictors was developed and presented as a nomogram (**Figure 2**). According to the score table, each variable has a corresponding score. We get the total score by calculating the score of each variable. Next, by plotting the total score on the probability scale, the ENE probability of lymph nodes can be estimated at the predicted risk points (**Figure 2**).

**Figure 3:**
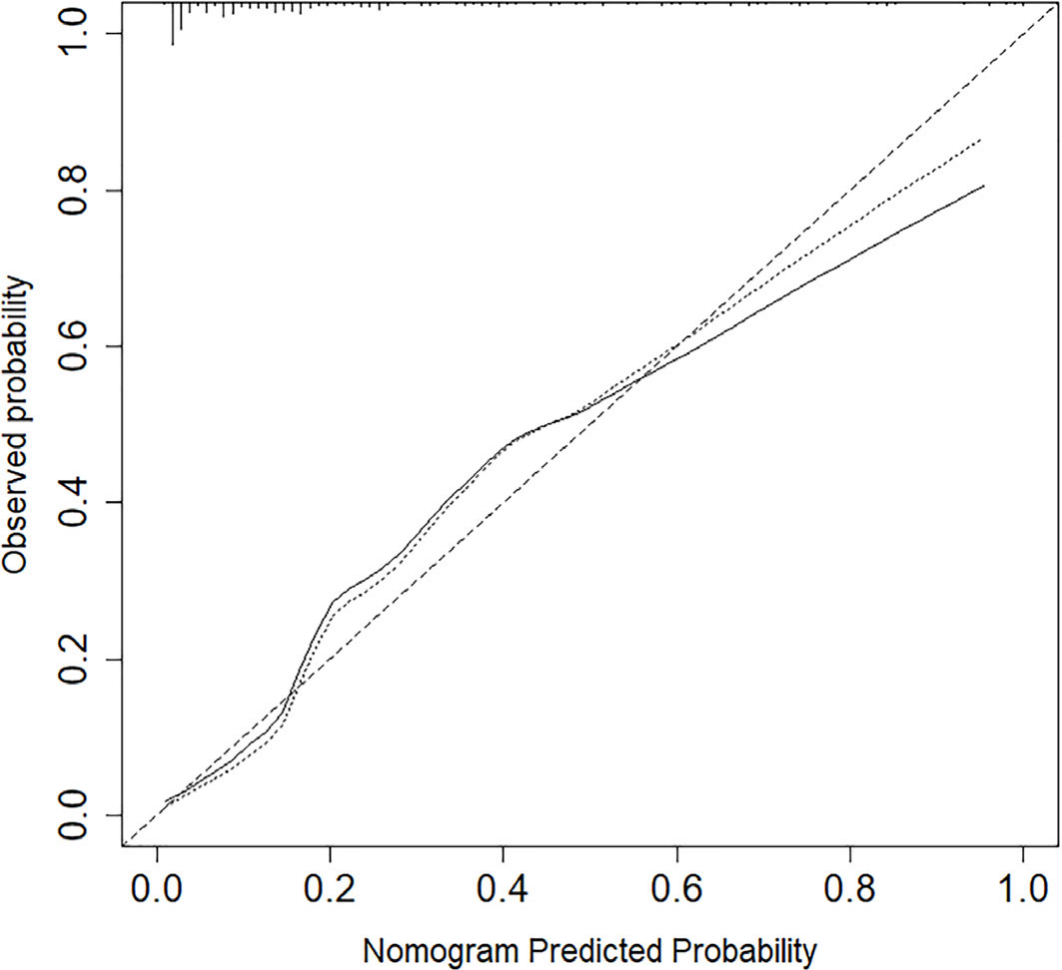
Nomogram calibration between the predicted risk and observed incidence. Calibration curves depict the calibration of models in terms of the agreement between the predicted risks of ENE and observed outcomes of ENE. The y-axis represents the actual ENE rate. The x-axis represents the predicted ENE risk. The diagonal dotted line represents a perfect prediction by an ideal model.

**Figure 4:**
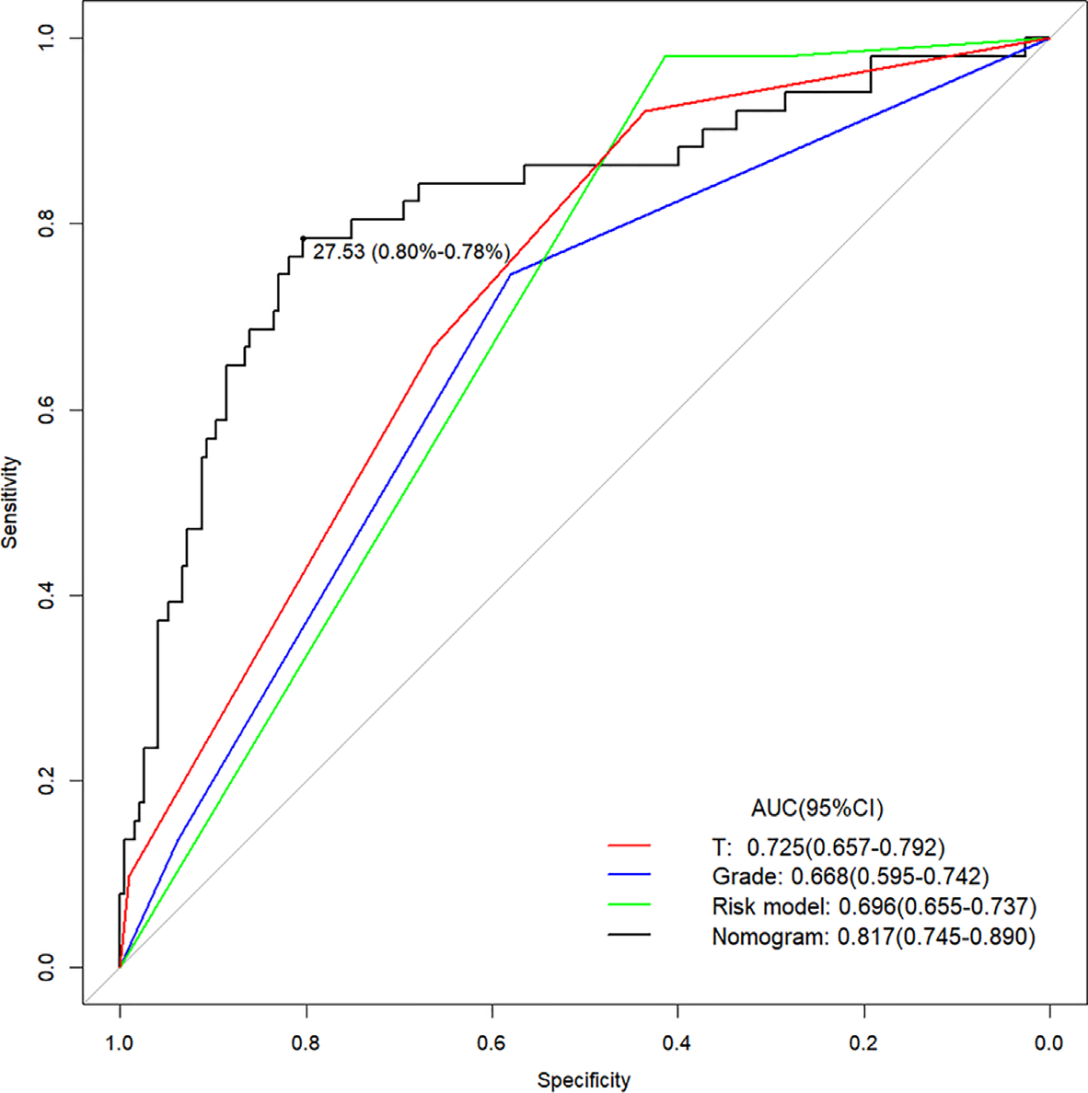
The Area Under Curve (AUC) of the prediction nomogram on T, Grade, Risk model and nomogram. T, tumor stage.

The calibration curve of the nomogram for the probability of lymph node ENE demonstrated good agreement between the prediction and observation in the cohort (**Figure 3**). The Hosmer-Lemeshow test yielded a nonsignificant statistic (P =0.340>0.05), which suggested that there was no departure from a perfect fit. The C-index for the prediction nomogram was 0.817 (95% CI, 0.745 to 0.890) for the cohort (**Figure 4**), which was confirmed to be 0.864 by bootstrapping validation.

**Figure 5:**
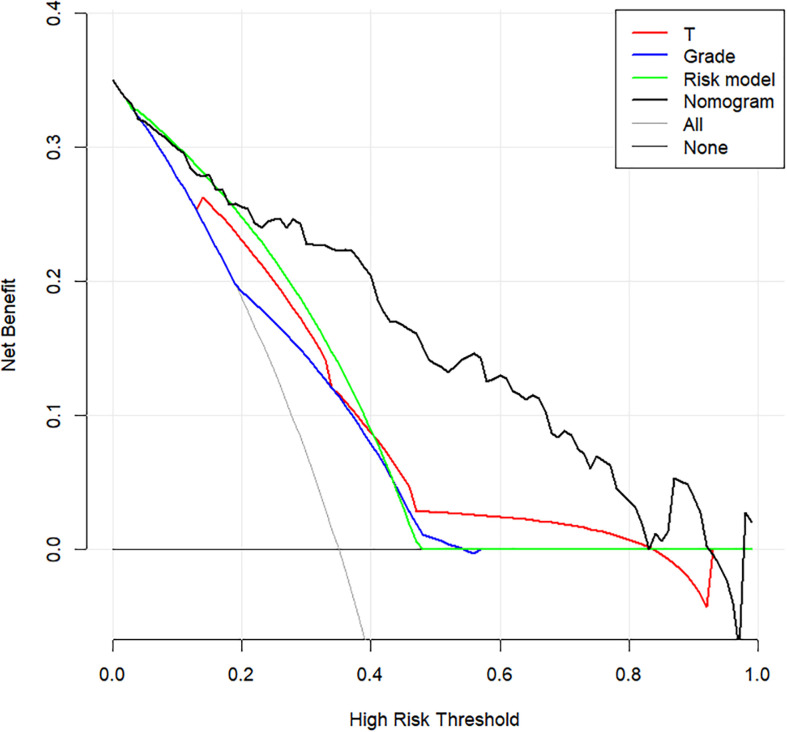
Decision curve analysis to assess the clinical usefulness of the nomogram, T stage, grade, Risk model and ENE. T stage, tumor stage; ENE, extranodal extension.

The prediction model after the addition of PLR and SCC-Ag is shown in **Table 4**. The highest C-index (0.817; 95% CI, 0.745 to 0.890) and the lowest AICs (180.034) was observed for the model with PLR and SCC-Ag integrated into the cohort. Comparison of Clinical Usefulness between Nomogram and some risk factors or EAU risk model, and the nomogram showed the best net benefit (**Figure 5**).

**Table 4:** Comparisons of different predictive models of Lymph Node ENE in Penile cancer.

Intercept and Variable	Clinical-laboratory nomogram		Model 2			Model 3		Model 4	
	Odds Ratio (95% CI)	P^a^	Odds Ratio (95% CI)		P^a^	Odds Ratio (95% CI)	P^a^	Odds Ratio (95% CI)	P^a^
Intercept		50							
SCC-Ag	1.088 (1.035-1.143)	0.001	NA	NA		1.095(1.042-1.152)	<0.001	NA	NA
PLR	1.013 (1.006-1.019)	<0.001	1.013 (1.007-1.02)	<0.0001		NA	NA	NA	NA
pT-stage	2.385( 1.488-3.823)	0.006	2.481 (1.574-3.912)	<0.0001		2.549 (1.628-3.991)	<0.0001	2.661(1.74-4.069)	<0.0001
LVI	3.077 (1.193-7.938)	0.017	4.976 (2.1-11.789)	<0.001		2.642 (1.067-6.539)	0.036	4.892 (2.19-10.925)	<0.001
C-index	0.817(0.745-0.890)		0.799 (0.724-0.874)			0.781(0.709-0.853)		0.640 (0.570-0.710)	
AIC	180.034		189.824			197.480		211.036	

A higher C-index indicates better discrimination and a lower AIC indicates superior model-fitting.

Clinical-laboratory nomogram, variables included, SCC-Ag, PLR, pT-stage, and LVI. Model 2, variables included, PLR, pT-stage, and LVI. Model 3, variables included, SCC-Ag, pT-stage, and LVI. Model 4, variables included, pT-stage, and LVI.

CI, Confidence Interval; OR, odds ratio; PLR, platelet-lymphocyte ratio; SCC-Ag, squamous cell carcinoma antigen; pT-stage, pathology tumor stage; LVI, Lymphovascular invasion. ^a^P values were calculated using Logistic regression model.

## Discussion

ENE is one of the most important predictors of unfavorable outcomes in PSCC and determines TNM staging and therapeutic options. However, there is no unified institutional clinical practice guidelines or established method for the diagnosis of ENE. We successfully developed and validated a predictive model, a new nomogram to predict lymph node ENE in PSCC patients. Here, we describe the first successful establishment of a prediction model for ENE. Incorporating laboratory and clinical factors into an easy-to-use nomogram for the prediction of lymph node ENE.

PSCC with lymph node ENE has a low survival rate. As early as 1987, Srinivas et al. (9) reported that lymph node metastasis-positive PSCC with ENE was related to a higher mortality than patients without lymph node ENE. Lughezzani et al. (10) identified ENE (OR, 8.01; P <.001) as a strong, independent predictor of PLNM. Niels et al. (11) reported that in patients without ENE, the five-year survival can be as high as 80% compared to a 5-year cancer-specific survival of 42% in patients with ENE.

In 2020, the NCCN guidelines recommended that PLND should be performed at the time or following ILND in the presence of ENE on final pathologic review (2). In addition, adjuvant external beam radiation therapy (EBRT) or chemoradiotherapy can be considered for patients with ENE. This means that patients with ENE are recommended to receive subsequent PLND and postoperative adjuvant therapy. Therefore, the prediction of lymph node ENE prior to ILND is important for selecting the most appropriate surgical procedure and postoperative adjuvant therapy. For patients with lower-risk or high-risk tumors who didn’t received immediate ILND, we recommend active surveillance and partially patients may experience an inguinal nodal recurrence during follow-up. Some patients underwent secondary inguinal lymph node dissection after primary surgery. For this patient group, if any patients that have predicted ENE according to their nomogram, we suggest 4 courses of neoadjuvant TIP and stable or responding disease should then undergo a PLND together with ILND and thereby avoid secondary procedures.

The pT-stage of the primary tumor is a strong predictor of high cancer-specific mortality (CSS) (12). Previous studies confirmed that patients with LVI seem to have systemic disease and is related to an addition risk of invasion and metastasis and was a significant independent predictor of a shorter OS (1, 2, 11, 12). As everyone knows, the infiltration of tumor cells into lymphatic vessels or blood vessels is the committed step of tumor diffusion.

A recent study (13, 14) investigated the prognostic value of the preoperative tumor marker SCC-Ag and systemic inflammatory factors in penile cancer. Interestingly, similar to our results, we found that SCC-Ag and PLR are highly correlated with the presence of ENE. SCC-Ag levels have been validated to predict LNM and have prognostic significance for disease-free survival(DFS) in patients with penile cancer treated with surgery (15). The predictive value of PLR has been investigated in various cancers (16, 17). The pretreatment PLR has been demonstrated to be a significant predictor in patients with cervical (18–20), colon, and colorectal cancer (21). The precise molecular mechanisms underlying the repercussion of PLR in PSCC remain unknown. Platelets, as a critical source of cytokines, bind FGF, PDGF, VEGF, and TGF-β family proteins, permitting plts to serve as a reservoir for secreted growth factors that promote tumorigenesis and the development of metastasis (22–24). Lymphocytes act a pivotal part in withstanding cancer cells by inducing cytotoxic cell death and inhibiting tumor cell proliferation and migration. Tumor infiltrating lymphocytes (TILs) are vital immune cells found in tumors, eligible for anti-tumor immune response (25). Taken together, PLR combined with the effects of platelets and lymphocytes may predict the presence of lymph node ENE.

The generalisability of these results suffer from several limitations. First, there was an inevitable selection bias, as the study was retrospectively designed. Secondly, imaging features were not included in our analysis. We see this investigation as an exploratory study and our aim is to provide clinicians with a good predictive tool which can serve as effective adjunctive tools with anatomic imaging, instead of contain imaging features. We believe that the present analysis or others which including imaging features variables will be important in future validation studies of larger and multicenter cohorts. Another limitation may be the smaller proportion of ENE-positive patients (22.6% [53/234]), although this study had a relatively large sample size. However, as our study included all patients who underwent bilateral ILND (including prophylactic ILND), the relatively small proportion who were ENE-positive could thus be explained. Fourth, we didn’t test our data with an independent external validation set.

## Conclusion

This study presents a clinicopathologic and laboratory-based nomogram that incorporates PLR, SCC-Ag, lymphovascular invasion (LVI), and pT-stage, which can be easily utilized to promote the individualized prediction of lymph node ENE in patients with PSCC.

## Data Availability Statement

The raw data supporting the conclusions of this article will be made available by the authors, without undue reservation.

## Ethics Statement

Written informed consent was obtained from the individual(s) for the publication of any potentially identifiable images or data included in this article.

## Author Contributions

HH had full access to all the data in the study and take responsibility for the integrity of the data and the accuracy of the data analysis.

Study conception and design: CW, ZL, and HH. Acquisition of data: CW, ZL, and HH. Analysis and interpretation of data: All. Drafting of the manuscript: CW and ZL. Critical revision of the manuscript for important intellectual content: All. Statistical analysis: CW, ZL, and HH. Obtaining funding: ZL and HH. Administrative, technical, or material support: FZ and HH. Supervision: HH. Other (specify): None. All authors contributed to the article and approved the submitted version.

## Funding

National Natural Science Foundation of China (Grant No.81772755, No.81902610). Science and Technology Planning Project of Shenzhen Municipality (CN) (JCYJ20190807145409328).

## Conflict of Interest

The authors declare that the research was conducted in the absence of any commercial or financial relationships that could be construed as a potential conflict of interest.

## Abbreviations

ENE, Extranodal extension; ILND, Inguinal lymph node dissection; PLR, Platelet-lymphocyte ratio; SCC-Ag, Squamous cell carcinoma antigen; PSCC, Penile squamous cell carcinoma; LNMs, Lymph node metastasis; PLND, Pelvic lymph node dissection; pT-stage, Pathologic tumor stage; LNI, Lymph node involvement; PNI, Perineural invasion; PLNM, Pelvic lymph node metastasis; LVI, Lymphovascular invasion.
