# Supplementary material for: Development and Validation of a Nomogram for the Prediction of Inguinal Lymph Node Metastasis Extranodal Extension in Penile Cancer
Source: Front Oncol. 2021 Jun 17;11:675565. doi: 10.3389/fonc.2021.675565 (PMC8247463; doi:10.3389/fonc.2021.675565)
Supplement: Supplementary file 1 [file Table_1.docx]

**Supplementary Table 1: 46 candidate lab tests.**

| Routine blood test | Coagulation function | Biochemistry test | Other |
| --- | --- | --- | --- |
| white blood cell count (WBC) | Prothrombin time (PT) | Kalium (K+) | Squamous cell carcinoma antigen (SCC-A) |
| Neutrophil%(NE%) | Prothrombin activity (PT%) | Natrium (Na+) | Blood type |
| Neutrophil# (NE#) | International normalized ratio (INR) | Chlorinum (Cl-) |  |
| Lymphocyte%(LY%) | Activated partial thromboplastin time (APTT) | Phosphorus (IP+++) |  |
| Lymphocyte#(LY#) | Thrombin time (TT) | Calcium (Ca) |  |
| Monocyte%(MO%) | Fibrinogen (Fbg) | Magnesium (Mg) |  |
| Monocyte#(MO#) | D-dimer (D-D) | Alanine aminotransferase (ALT) |  |
| Eosinophil%(EO%) | Fibrinogen Degradation Products (FDP) | Aspartate aminotransferase (AST) |  |
| Eosinophil#(EO#) |  | Aspartate/Alanine aminotransferase (AS/AL) |  |
| Basophil%(BA%) |  | Alkaline phosphatase (ALP) |  |
| Basophil#(BA#) |  | γ-glutamyl transpeptadase (GGT) |  |
| Red blood cell count(RBC) |  | Lactate dehydrogenase (LDH) |  |
| Hemoglobin(HGB) |  | Total protein (TP) |  |
| Platelet count(PLT) |  | Albumin (ALB) |  |
| Platelet-to-lymphocyte ratio (PLR) |  | Globulin (GLOB) |  |
| Neutrophil-to-lymphocyte ratio (NLR) |  | Albumin/Globulin (A/G) |  |
|  |  | Urea (UREA) |  |
|  |  | Creatinine (CRE) |  |
|  |  | Uric acid (UA) |  |
|  |  | C reactive protein (CRP) |  |
